# Selective Probiotic Treatment Positively Modulates the Microbiota–Gut–Brain Axis in the BTBR Mouse Model of Autism

**DOI:** 10.3390/brainsci12060781

**Published:** 2022-06-14

**Authors:** Angela Pochakom, Chunlong Mu, Jong M. Rho, Thomas A. Tompkins, Shyamchand Mayengbam, Jane Shearer

**Affiliations:** 1 Department of Biochemistry and Molecular Biology, Cumming School of Medicine, University of Calgary, Calgary, AB T2N 4N1, Canada; chunlong.mu1@ucalgary.ca (C.M.); jshearer@ucalgary.ca (J.S.); 2 Departments of Neurosciences, Pediatrics and Pharmacology, University of California San Diego (UCSD), La Jolla, CA 92093, USA; jrho@health.ucsd.edu; 3 Lallemand Inc., Lallemand Bio-Ingredients, Montreal, QC H1W 2N8, Canada; ttompkins@lallemand.com; 4 Department of Biochemistry, Memorial University of Newfoundland, St. John’s, NL A1C 5S7, Canada; smayengbam@mun.ca; 5 Faculty of Kinesiology, University of Calgary, Calgary, AB T2N 4N1, Canada; 6 Alberta Children’s Hospital Research Institute, Cumming School of Medicine, University of Calgary, Calgary, AB T2N 4N1, Canada

**Keywords:** probiotics, autism, gut microbiota, metabolism, mitochondria

## Abstract

Recent studies have shown promise for the use of probiotics in modulating behaviour through the microbiota–gut–brain axis. In the present study, we assessed the impact of two probiotic strains in mitigating autism-related symptomology in the BTBR *T^+^ Itpr3^tf^*/J mouse model of autism spectrum disorder (ASD). Male juvenile BTBR mice were randomized into: (1) control, (2) *Lr* probiotic (1 × 10^9^ CFU/mL *Lacticaseibacillus rhamnosus* HA-114), and (3) *Ls* probiotic groups (1 × 10^9^ CFU/mL *Ligilactobacillus salivarius* HA-118) (n = 18–21/group), receiving treatments in drinking water for 4 weeks. Gut microbiota profiling by 16S rRNA showed *Lr*, but not *Ls* supplementation, to increase microbial richness and phylogenetic diversity, with a rise in potential anti-inflammatory and butyrate-producing taxa. Assessing serum and brain metabolites, *Lr* and *Ls* supplementation produced distinct metabolic profiles, with *Lr* treatment elevating concentrations of potentially beneficial neuroactive compounds, such as 5-aminovaleric acid and choline. As mitochondrial dysfunction is often observed in ASD, we assessed mitochondrial oxygen consumption rates in the prefrontal cortex and hippocampus. No differences were observed for either treatment. Both *Lr* and *Ls* treatment reduced behavioural deficits in social novelty preference. However, no changes in hyperactivity, repetitive behaviour, and sociability were observed. Results show *Lr* to impart positive changes along the microbiota–gut–brain axis, exhibiting beneficial effects on selected behaviour, gut microbial diversity, and metabolism in BTBR mice.

## 1. Introduction

Autism spectrum disorder (ASD) is a common neurodevelopmental disorder that is characterized by impairments in communication and social interaction, along with stereotyped and repetitive behaviours [1]. ASD is one of the fastest-growing neurodevelopmental disorders in North America, with an estimated prevalence of 1 in 44 children [2]. Due to its heterogeneous nature, the etiology of ASD has been difficult to define. Several theories have been proposed pertaining to the potential causes of ASD, which include environmental factors, genetic defects, abnormalities in neuronal function, and metabolic shifts [3,4,5].

More recently, the relationship between ASD and gastrointestinal (GI) distress has gained considerable attention, with GI symptoms presenting in 46% to 84% of autistic children [6]. Furthermore, a strong correlation has been found between GI symptoms and autism severity, suggesting a potential relationship between the gut and brain dysfunction in affected individuals [7]. GI disorders are believed to be linked to intestinal dysbiosis, which is often defined as a loss of microbial diversity combined with an imbalance of commensal to pathogenic bacteria [8,9]. 

Several studies have demonstrated gut microbial dysbiosis in children with ASD [10], a phenotype thought to be driven by poor diet diversity and altered stool consistency [11]. There is accumulating evidence to indicate that developmental changes to the gut microbiota can lead to long-term behavioural outcomes in both animal models and human patients [12,13,14]. As such, it is largely unknown whether the microbiota is a modifiable risk factor for the development and progression of ASD. Given this, early intervention with probiotics to counteract microbial dysbiosis has gained significant attention as a potential treatment for ASD symptoms. 

The International Scientific Association for Probiotics and Prebiotics defines probiotics as “live microorganisms that, when administered in adequate amounts, confer a health benefit on the host” [15]. Probiotic supplementation has been used to relieve symptoms of GI disorders by restoring bacterial microbiota and affecting GI function through various mechanisms [16]. A recent study, surveying 210 children with ASD, found that 37% of the participants regularly consumed probiotics [17]. While probiotic intervention has exhibited some success in improving ASD and GI symptomology, there is a paucity of data regarding the potential mechanisms of probiotic action in this population—a limitation to developing targeted formulations for ASD individuals. The answer may be found in the concept of ‘psychobiotics’, a term coined by Dinan and colleagues [18], which describes a class of probiotics that produce neuroactive compounds to act upon the microbiota–gut–brain axis. The consequent activity of these neuroactive compounds can lead to changes in behaviour, gut function (e.g., intestinal epithelial permeability/leakiness), and energy metabolism [19,20,21]. With respect to the latter, these compounds can directly modulate mitochondrial respiration, the main process that catalyzes ATP production in the body and brain. As mitochondrial dysfunction is present in many ASD individuals [22], mitochondria represent another potential therapeutic target for gut microbiota-derived metabolites. 

The aim of the present study was to investigate the psychobiotic potential of two probiotic strains in a mouse model of ASD, *Lacticaseibacillus rhamnosus* HA-114 and *Ligilactobacillus salivarius* HA-118 (both previously categorized under the *Lactobacillus* genus). To gain better insight into their mechanisms of action, alterations in ASD-related behaviours, gut microbiota, and metabolism (i.e., systemic and cellular) were evaluated. We hypothesized that probiotic administration would ameliorate the core behavioural deficits associated with ASD by mitigating intestinal dysbiosis and improving metabolic parameters. 

## 2. Materials and Methods

### 2.1. Animals

All experimental protocols were approved by the Life and Environmental Sciences Animal Care Committee at the University of Calgary and followed the guidelines on ethical animal use set out by the Canadian Council on Animal Care (Protocol #AC17-0217). The BTBR *T*^+^ *Itpr3^tf^*/J (BTBR) inbred strain was purchased from The Jackson Laboratory (Bar Harbor, ME, USA) and a colony was maintained for the duration of the study. Male BTBR mice were weaned on postnatal day 21 (P21) and housed with 2–4 littermates in standard plastic cages with woodchip bedding, paper nesting material, and one enrichment object. Animals were kept in temperature- and humidity-controlled rooms maintained under a 12:12 h light/dark cycle at the University of Calgary Health Sciences Animal Resource Centre facility. The mice were provided with a standard laboratory chow diet (Pico-Vac^®^ Mouse Diet 20) and probiotic treatment (i.e., water (control), *L. salivarius* HA-118, and *L. rhamosus* HA-114) *ad libitum.* Body mass measurements were taken at baseline (P21), 2 weeks (P35), and 4 weeks (P49) of probiotic treatment.

### 2.2. Probiotic Administration

*Ligilactobacillus salivarius* HA-118 (*Ls*) and *Lacticaseibacillus rhamnosus* HA-114 (*Lr*) strains were provided by Lallemand Health Solutions (Mirabel, QC, Canada). Both strains were administered to mice through autoclaved drinking water to reach a concentration of 1 × 10^9^ CFU/mL. Control mice were provided with equivalent volumes of drinking water without probiotic strains. Fresh solutions were provided every 48 h. Probiotic treatment was provided for 4 weeks prior to sacrifice (i.e., anaesthesia with 5% isoflurane followed by decapitation) and sample collection.

### 2.3. Behavioural Assessments

BTBR mice underwent behavioural testing at 6 weeks of age, a developmental period associated with the mid-adolescence stage in rodents [23]. All behavioural assessments were completed in the light phase and mice were habituated to the testing environment for at least 30 min before each test without prior handling. 

The testing battery was targeted towards the core behavioural deficits associated with ASD. Hyperactivity and anxiety-like behaviours were measured with the open field test, where mice were placed in the centre of a clean arena and allowed to explore freely for a period of 10 min [24]. The open field test assesses anxiolytic behaviour in rodents based on the phenomena of photophobicity, an innate behaviour in which mice prefer to stay closer to walls and avoid lit open spaces (i.e., centre of arena), as they are nocturnal animals [25]. Repetitive, stereotyped behaviour was assessed through self-grooming activity, which was measured as previously described [26]. Further evaluation of repetitive behaviours was conducted with the marble-burying test, in which the number of marbles buried under bedding within a 5 min period was measured [27]. The marble-burying test assesses repetitive, stereotyped behaviour in ASD by utilizing the tendency of mice to dig in natural (i.e., burrows, escape tunnels) and standard cage (i.e., bedding) environments [27]. Lastly, the three-chamber test was performed to assess sociability and social novelty interactions, following previously established experimental protocols [24], with the use of sex- and age-matched controls. Sociability is defined by the time spent interacting with a social (i.e., mouse) versus non-social (i.e., cup) stimulus as a measure of social affiliation/motivation [28]. Social novelty preference is determined by the time spent interacting with a familiar versus a novel conspecific, measuring social memory and novelty [28].

### 2.4. Fecal Microbial Analysis

Fecal samples were collected at three timepoints: baseline, 2 weeks, and 4 weeks of probiotic administration. Samples were stored at −80 °C following collection. Bacterial genomic DNA was extracted from the 4-week fecal samples as per protocol (DNeasy Powersoil Pro Kit, QIAGEN, Hilden, Germany). All samples were diluted to a concentration of 20 ng/μL with nuclease-free water and kept at −20 °C for further analysis. The V3–V4 region of the microbial 16S rRNA gene was targeted for high-throughput sequencing using the MiSeq Illumina platform at the University of Calgary Centre for Health Genomics and Informatics (https://cumming.ucalgary.ca/research/cat/health-genomics/home, accessed on 13 April 2022). PCR amplification protocols and library preparation were completed as described by Illumina [29]. Sequences were denoised and filtered using the QIIME 2 2021.10 software package [30]. Taxonomic classification was completed at the amplicon sequence variant (ASV) level, using SILVA 138 as a reference database. A trained classification model, created with the QIIME 2 q2-sample-classifier function, was used to determine differences in relevant ASVs between groups. Further pairwise comparisons between groups were used to confirm the differential abundance results. Principal coordinates analysis using Unifrac distances was used to visualize differences in microbial community composition between groups [31]. Quantification of multivariate community-level differences between groups was completed using a permutational multivariate analysis of variance (PERMANOVA) [32]. Functional annotations were then predicted using the PICRUSt2 software [33].

### 2.5. Cytokine Analysis

Serum was collected at sacrifice (P49) and diluted 2-fold with PBS (pH~7.5). Samples underwent a Mouse Cytokine 10-Plex Discovery Assay^®^ Array (Eve Technologies Corporation, Calgary, AB, Canada) to assess the following cytokines: granulocyte-macrophage colony-stimulating factor (GM-CSF), interferon gamma (IFNγ), interleukin 1 beta (IL-1β), interleukin 2 (IL-2), interleukin 4 (IL-4), interleukin 6 (IL-6), interleukin 10 (IL-10), interleukin 12p70 (IL-12p70), monocyte chemoattractant protein-1 (MCP-1), tumour necrosis factor alpha (TNFα). IFNγ levels were below detection limits for >60% of samples and were therefore excluded from analysis. Results are reported in pg/mL.

### 2.6. Metabolic Assessment

Blood glucose and ketone levels were assessed at sacrifice (P49) using a FreeStyle Precision Neo meter (Abbott Laboratories, Abbott Park, IL, USA). Quadrupole time-of-flight (Q-TOF) liquid chromatography–mass spectrometry (LC-MS) was conducted to perform untargeted metabolomic profiling of serum and prefrontal cortex tissue samples of BTBR mice. Sample preparation was performed as previously described [34]. Briefly, 200 μL of ice cold 100% HPLC/MS-grade Methanol (MilliporeSigma, Darmstadt, Germany) was added to 50 μL of serum and vortexed for 60 s to form a homogenous solution. Following centrifugation, the supernatant was collected and dried in a Savant SpeedVac SVC-100 (Hyderabad, India). 100 μL of 50% methanol, diluted with HPLC/MS-grade Water (MilliporeSigma, Darmstadt, Germany), was used to reconstitute the dried samples, which were then centrifuged (14,000× *g*; 4 °C) for 15 min. 90 μL of flow-through was collected and added into a LC/MS glass vial with a glass insert. 

For tissue, 10 mg of prefrontal cortex was homogenized with 200 μL of an ice-cold 2:1 (*v*/*v*) methanol/water solution and homogenization beads. The solution was sonicated in an ice bath for 5 min, stored at −20 °C for 1 hr, and then underwent centrifugation (14,000× *g*; 4 °C) for 15 min. From the time of supernatant collection and drying of samples, the protocol for prefrontal-cortex-tissue preparation follows the serum-preparation protocol mentioned above. 2 μL of extracts were injected into an Agilent 6550 iFunnel QTOF LC-MS system (Agilent Technologies Canada Inc., Mississauga, ON, Canada) with a flow rate of 0.4 mL/min. A HSS T3 chromatographic column (C_18_, 100 mm × 2.1 mm, 1.8 μm particle size) maintained at 40 °C was used for liquid chromatography. A gradient elution method was applied using water and acetonitrile, both modified with 0.1% formic acid, as solvents. Mass spectrometry was used to detect both aqueous and nonaqueous (i.e., lipid) compounds with a mass rage of 50–1000 *m*/*z*. ProteoWizard 3.0 (Palo Alto, CA, USA) was used to convert raw Agilent data to mzXML files for further LC-MS data analysis. Open-sourced programs XCMS online (https://xcmsonline.scripps.edu/, accessed on 25 November 2021) and MetaboAnalyst V5.0 (https://www.metaboanalyst.ca/, accessed on 25 November 2021) were used for LC-MS data preprocessing and statistical analysis [35,36]. Metabolite identification was performed using the METLIN database (http://metlin.scripps.edu/, accessed on 25 November 2021), the Human Metabolome Database (HMDB V5.0), and LIPID MAPS [37,38]. Variable importance in projection (VIP) scores were obtained from partial least-squares discriminant analysis (PLS-DA) [39]. PLS-DA allows for the visualization of group structure and separation from spectral features that contribute to between-group variability [40]. A cross-validation analysis of variance (CV-ANOVA) was conducted to assess the reliability of the PLS-DA model with SIMCA-P 13 (Umetrics AB, Umeå, Sweden) [41]. An FDR-adjusted *p*-value < 0.05 and VIP score > 2 was used to define discriminant metabolites. A Tukey’s post hoc test was completed to determine differential abundance of metabolites between groups.

### 2.7. Mitochondrial Energetics

High-resolution respirometry in prefrontal cortex and hippocampus tissue was measured using the Oroboros Oxygraph-2K system (O2K; Oroboros Instruments, Innsbruck, Austria) [42,43]. Briefly, 2 mg of fresh tissue was placed into chambers filled with 2.0 mL of MiR05 (37 °C) and manual titrations of substrates were injected into the chamber using Hamilton syringes. Titrations were performed in the following order: saponin, PMG (pyruvate, malate, glutamate), adenosine diphosphate, cytochrome C, succinate, FCCP (carbonyl cyanide p-trifluoro-methoxyphenyl hydrazone), rotenone, and antimycin. Specific substrate concentrations and functions are described in Supplementary Table S1. Real-time data acquisition and subsequent analysis of high-resolution respirometry were completed using DatLab 7 software (Oroboros Instruments, Innsbruck, Austria).

### 2.8. Statistical Analysis

All statistical analysis was performed on the GraphPad Prism (v 9.2.0) software (GraphPad Software Inc, San Diego, CA, USA), unless explicitly stated. Outliers were removed using ROUT analysis [44]. A one-way analysis of variance (ANOVA) followed by a Tukey’s multiple comparisons test was used to determine differences between multiple groups, given that they passed the Shapiro–Wilk test of normality. Multiple comparisons were corrected by controlling the false discovery rate (FDR) using the Benjamini–Hochberg procedure where applicable [45]. An FDR of <0.05 was considered statistically significant. Individual comparisons between specific groups were completed with an unpaired *t*-test. A Welch’s correction was applied to the unpaired *t*-test if the variance among both populations differed significantly. If normality tests were not passed, the appropriate nonparametric tests were applied to the datasets (i.e., Mann–Whitney U test, Kruskal–Wallis test). Pearson correlation analysis was completed to determine potential relationships between microbial composition, discriminant metabolites, mitochondrial respiration, and behavioural changes.

## 3. Results

### 3.1. Effects of Probiotics on ASD-Related Behaviours

**Figure 1:**
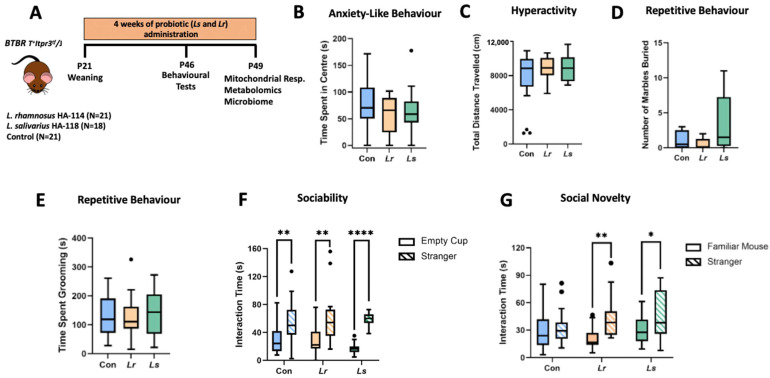
Effects of probiotic treatment on behavioural deficits associated with ASD in BTBR mice. (**A**) A schematic diagram of the experimental design. (**B**–**F**) A behavioural test battery was performed looking at nonsocial anxiety behaviours (N = 17–21) (**B**), hyperactivity (N = 16–21) (**C**), repetitive behaviours (N = 11–20) (**D**,**E**) and sociability/social novelty (N = 14–19) (**F**,**G**) between groups. Con—Control; *Lr*—*L. rhamnosus* HA-114; *Ls*—*L. salivarius* HA-118. Boxes extend from the 25th to 75th percentiles with the whiskers representing the furthest point that is within 1.5 times the interquartile range (IQR). * *p* < 0.05; ** *p* < 0.01, **** *p* < 0.0001.

Time spent in the centre of the open field was used as a proxy for anxiety-like behaviours in rodents [46]. There were no statistically significant differences in the amount of time spent in the centre of the open field between groups (*p* = 0.675) (Figure 1B). Hyperactivity was measured through locomotor activity (i.e., total distance travelled) in the open field test. No significant differences in hyperactivity were found between groups (*p* = 0.600) (Figure 1C). Marble burying and self-grooming are commonly used as a measure of repetitive behaviour in rodents [27]. There were no significant differences in marble burying (*p* = 0.165) or self-grooming (*p* = 0.760) between groups (Figure 1D,E). The three-chamber task has shown strong face validity and reliability for assessing deficits in social interaction in rodent models of ASD [47]. During the sociability session, all groups showed a significant preference for the social stimuli over the non-social stimuli (Figure 1F). In the social novelty session, only the *Lr* (*p* = 0.002) and *Ls* (*p* = 0.045) treatment groups, not the control group (*p* = 0.542), showed a preference for the novel social stimulus over the familiar social stimulus (Figure 1G). As such, both *Lr* and *Ls* treatments were able to reduce deficits in social novelty preference compared to control BTBR mice.

### 3.2. Effects of Probiotics on Fecal Microbiota Composition and Function

**Figure 2:**
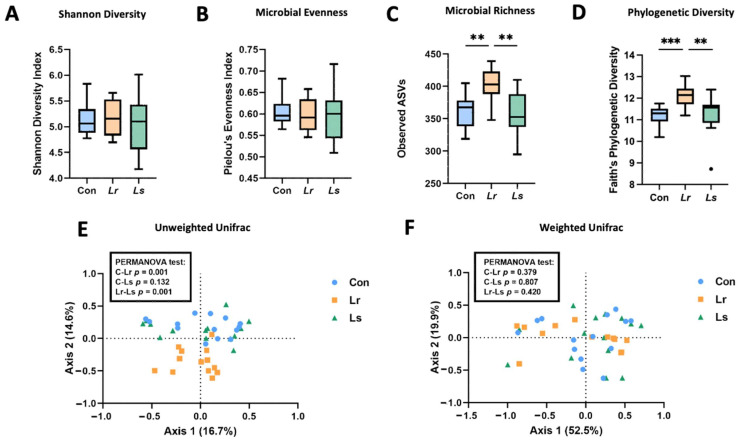
α- and β-diversity metrics of fecal microbiota following probiotic treatment. (**A**–**D**) Microbial richness, evenness, and phylogenetic diversity as measured by various α-diversity parameters (i.e., Shannon Diversity (**A**), Pielou’s Evenness (**B**), Observed ASVs (**C**), and Faith’s Phylogenetic Diversity (**D**) indices). (**E**,**F**) Principal Coordinate Analysis (PCoA) plot of unweighted and weighted Unifrac distances as a measure of microbial community structure. N = 13–14 mice/group. Con—Control; *Lr*—*L. rhamnosus* HA-114; *Ls*—*L. salivarius* HA-118. Boxes extend from the 25th to 75th percentiles with the whiskers representing the furthest point that is within 1.5 times the IQR. ** *p* < 0.01, *** *p* < 0.001.

To assess the effect of probiotic strains *L. rhamnosus* HA-114 and *L. salivarius* HA-118 on the BTBR gut microbiota, 16S rRNA gene sequencing was completed on fecal samples following 4 weeks of treatment. While there were no significant changes in Shannon diversity (*p* = 0.804, Figure 2A) and microbial evenness (*p* = 0.876, Figure 2B), the *Lr* group exhibited significantly higher microbial richness and phylogenetic diversity compared to the control and *Ls* groups, as measured by the Observed ASVs (*p* < 0.01, Figure 2C) and Faith’s PD (*p* < 0.01, Figure 2D) indices, respectively. Distinct microbial community structures across groups were assessed using UniFrac distances (Figure 2E). A pairwise PERMANOVA determined that the *Lr* group had significantly distinct microbial clustering compared to the control (*p* = 0.001) and *Ls* (*p* = 0.001) groups using unweighted Unifrac distances (Figure 2E). No significant differences in community structure were observed between groups (*p* = 0.614) using weighted Unifrac distances (Figure 2F). As unweighted Unifrac is more sensitive to shallow branches on the phylogenetic tree, our results indicate that microbial changes were observed in bacteria of lower abundance in our samples, which are often not detected using weighted Unifrac [48]. These results suggest that *Lr* administration may improve gut dysbiosis by increasing microbial diversity, as measured by α- and β-diversity metrics.

**Figure 3:**
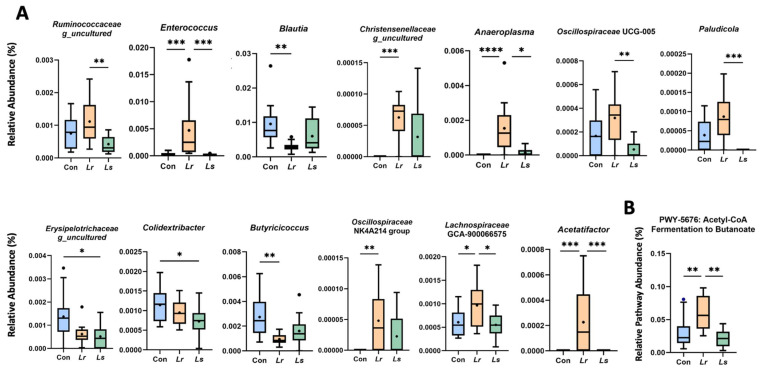
Differentially abundant taxa and functional predictions following probiotic treatment**.** (**A**) Box plots of significantly different genera between groups. Genera are listed in order of decreasing feature importance (right to left), as determined by a trained classification model. (**B**) Metagenomic analysis of butanoate metabolism evaluated by PICRUSt2. N = 10–14 mice/group. Con—Control; *Lr*—*L. rhamnosus* HA-114; *Ls*—*L. salivarius* HA-118. Means are indicated by the black circles located within the box. The box extends from the 25th to 75th percentiles with the whiskers representing the furthest point that is within 1.5 times the IQR.* *p* < 0.05; ** *p* < 0.01, *** *p* < 0.001, **** *p* < 0.0001.

The relative abundances of the *Enterococcus*, *Anaeroplasma*, *Lachnospiraceae* GCA-900066575, and *Acetatifactor* genera were significantly increased with *Lr* administration compared to both control and *Ls* groups (Figure 3A). Specifically, *Lr* administration promoted the abundance of *Christensenellaceae* genus_uncultured and *Oscillospiraceae* NK4A214, while suppressing populations of *Blautia* and *Butyricicoccus* compared to the control group (Figure 3A). In comparison to the *Ls* strain, *Lr* supplementation resulted in increases in *Ruminococcaceae* genus_uncultured, *Oscillospiraceae* UCG-005, and *Paludicola* (Figure 3A). *Ls* administration suppressed populations of *Erysipelotrichaceae* genus_uncultured, and *Colidextribacter* compared to controls, with no increases in other taxa compared to both control and *Lr* groups (Figure 3A). These results are indicative of differences in bacterial community composition between groups that are specific to particular taxa.

As certain taxa in our differential abundance analysis were associated with butyrate production, we assessed the functional potential of our bacterial groups in butanoate (i.e., butyrate) metabolism using PICRUSt2. We found a significant increase in the abundance of MetaCyc pathway *PWY-5676* in the *Lr* group compared to the control and *Ls* groups (*p* < 0.01, Figure 3B). *PWY-5676* encompasses the fermentation of acetyl-CoA to butanoate, a major pathway in butyrate synthesis facilitated by gut microbes.

### 3.3. Cytokine Profiles

**Figure 4:**
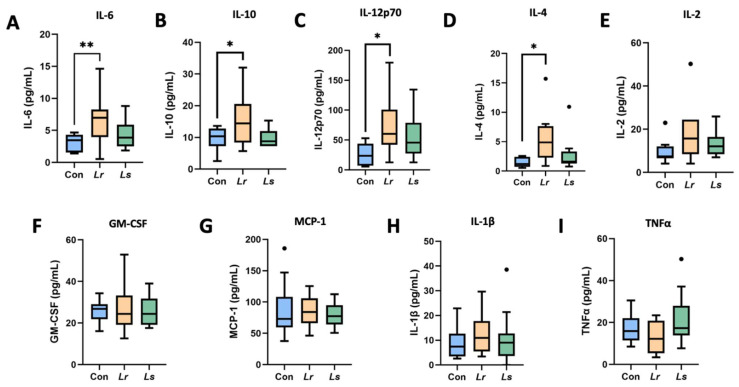
Panel of serum cytokine levels following probiotic treatment. Detected concentrations of IL-6 (**A**), IL-10 (**B**), IL-12p70 (**C**), IL-4 (**D**), IL-2 (**E**), GM-CSF (**F**), MCP-1 (**G**), IL-1β (**H**), and TNFα (**I**) in serum between groups. N = 10–14 mice/group. Con—Control; *Lr*—*L. rhamnosus* HA-114; *Ls*—*L. salivarius* HA-118. The box extends from the 25th to 75th percentiles with the whiskers representing the furthest point that is within 1.5 times the IQR. * *p* < 0.05; ** *p* < 0.01.

Cytokine profiling determined that IL-6 (*p* = 0.004), IL-10 (*p* = 0.046), IL-12p70 (*p* = 0.019) and IL-4 (*p* = 0.016) were significantly increased in the *Lr* group compared to the control group (Figure 4A–D). No significant differences were found between groups for IL-2 (*p* = 0.103), GM-CSF (*p* = 0.852), MCP-1 (*p* = 0.925), IL-1β (*p* = 0.746), TNFα (*p* = 0.162) (Figure 4E–I).

### 3.4. Blood and Serum Metabolic Alterations following Probiotic Treatment

**Figure 5:**
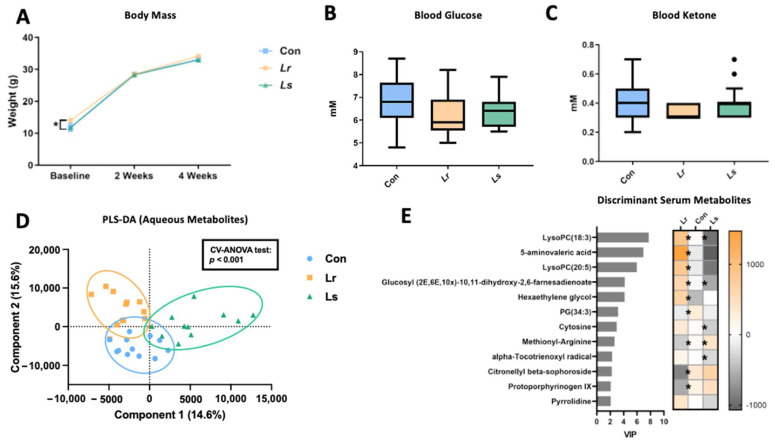
Effect of probiotic treatment on body mass and systemic metabolism. (**A**) Body weight of mice recorded over the 4-week treatment period. Significant differences at baseline were observed between *Lr* and *Ls* groups. (**B**,**C**) Blood glucose and ketone levels measured at sacrifice (N = 17–21) (**D**) PLS-DA analysis of aqueous serum metabolites. (**E**) Discriminant serum metabolites with *p* < 0.05 and VIP > 2. Significant differences (*p* < 0.05) in the abundance of metabolites in the *Lr* and *Ls* groups compared to controls determined by Tukey’s post hoc analysis are indicated by ‘*’ on the heatmap. Boxes extend from the 25th to 75th percentiles with the whiskers representing the furthest point that is within 1.5 times the interquartile range (IQR). N= 11–12 mice/group. Con—Control; *Lr*—*L. rhamnosus* HA-114; *Ls*—*L. salivarius* HA-118. * *p* < 0.05.

We assessed the effect of probiotic administration on systemic metabolism through various measures. No changes were observed in body mass (*p* = 0.075), blood glucose (*p* = 0.093), and blood ketones (*p* = 0.355) between groups following 4 weeks of treatment (Figure 5A–C). Metabolomics profiling of serum samples revealed distinct metabolite composition among groups, as determined by PLS-DA analysis (*p* < 0.001) (Figure 5D). Assessing discriminant metabolites (*p* < 0.05, VIP > 2), *Lr* administration significantly altered the relative concentrations of several lipid and lipid-like molecules, with increases in lysophosphatidylcholine (LysoPC), hexaethylene glycol, and glucosyl (2E,6E,10×)-10,11-dihydroxy-2,6-farnesadienoate, as well as decreases in phosphatidylglycerol (PG) and citronellyl beta-sophoroside compared to controls (Figure 5E). Evaluating concentrations of neuroactive compounds, *Lr* administration increased levels of 5-aminovaleric acid (5-AV) (*p* = 0.003), a GABA receptor agonist, and decreased levels of protoporphyrinogen IX (*p* = 0.007), a potential neurotoxin and metabotoxin [49,50]. Conversely, *Ls* administration resulted in an overall metabolic profile that was mainly opposite of what was observed in the *Lr*, with only 5 of 12 discriminant metabolites showing divergent responses compared to controls including LysoPC, glucosyl (2E,6E,10×)-10,11-dihydroxy-2,6-farnesadienoate and methionyl-arginine (Figure 5E).

### 3.5. Brain Metabolomic Responses to Probiotic Treatment

**Figure 6:**
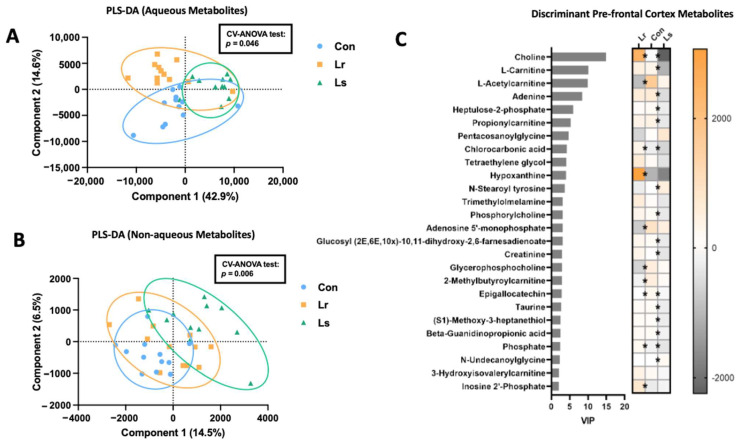
Effect of probiotic treatment on prefrontal cortex metabolism. (**A**) PLS-DA analysis of aqueous PFC metabolites. (**B**) PLS-DA analysis of nonaqueous PFC metabolites. (**C**) Discriminant PFC metabolites with *p* < 0.05 and VIP > 2. Significant differences (*p* < 0.05) in the abundance of metabolites in the *Lr* and *Ls* groups compared to controls are indicated by ‘*’ on the heatmap. Boxes extend from the 25th to 75th percentiles with the whiskers representing the furthest point that is within 1.5 times the interquartile range (IQR). N= 11–12 mice/group. Con—Control; *Lr*—*L. rhamnosus* HA-114; *Ls*—*L. salivarius* HA-118.

High-resolution metabolomics profiling of prefrontal cortex tissue was completed to further evaluate potential gut–brain-axis mediators. PLS-DA revealed distinct compositions of aqueous (*p* = 0.046) and nonaqueous (*p* = 0.007) metabolites between groups (Figure 6A,B). Probiotic administration resulted in overall decreased concentrations of carnitines compared to the control group, with *Lr* groups displaying lower levels of L-acetylcarnitine and 2-methylbutyroylcarnitine, and *Ls* groups presenting lower levels of L-carnitine and propionylcarnitine (*p* < 0.05, Figure 6C). Evaluating metabolites associated with CNS function, *Lr* administration resulted in increases of choline (*p* < 0.001) and inosine 2′-phosphate (*p* = 0.001), as well as reductions in adenosine 5-monophosphate (*p* = 0.004) (Figure 6C). Within the same category, *Ls* administration resulted in decreased concentrations of choline (*p* < 0.001), taurine (*p* = 0.012), and beta-guanidinopropionic acid (*p* = 0.012).

Overall, *Lr* and *Ls* administration produced distinct metabolic profiles, in both serum and the brain, suggesting changes in lipid metabolism, energy metabolism (i.e., carnitine), and the production of specific neuroactive compounds, which may be associated with behavioural alterations.

### 3.6. Brain Mitochondrial Respiration in Response to Probiotics

**Figure 7:**
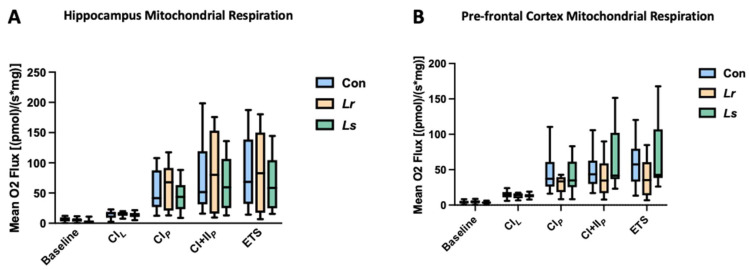
Effect probiotic treatment on mitochondrial respiration in the brain. Mitochondrial respiration rates were assessed in the hippocampus (**A**) and prefrontal cortex (**B**) of BTBR mice undergoing Lr, Ls, and control treatments. N = 10–16 mice/group. Abbreviations: Con—Control; *Lr*—*L. rhamnosus* HA-114; *Ls*—*L. salivarius* HA-118; CI*_L_*—Proton Leak Respiration in Complex I; CI*_P_*—Phosphorylation Respiration in Complex I; CI+II*_P_*—Phosphorylation Respiration in Complex I+II; ETS—Maximal Respiratory Capacity in the Electron Transport System. The box extends from the 25th to 75th percentiles with the whiskers representing the furthest point that is within 1.5 times the IQR.

A high prevalence of mitochondrial dysfunction is often observed in ASD individuals, with studies suggesting around 30–50% of cases exhibiting biomarkers for abnormal mitochondrial function [22]. To determine the effect of probiotic administration on mitochondrial metabolism, oxygen respiration rates were measured from mitochondria in the prefrontal cortex and hippocampus using the Oroboros Oxygraph-2k high-resolution respirometry system. No significant differences in mitochondrial oxygen consumption rates were observed between groups in the hippocampus and prefrontal cortex (Figure 7A,B). Overall, these results suggest that probiotic administration was not sufficient to alter mitochondrial respiration in the brain of BTBR mice.

### 3.7. Correlation Analysis

**Figure 8:**
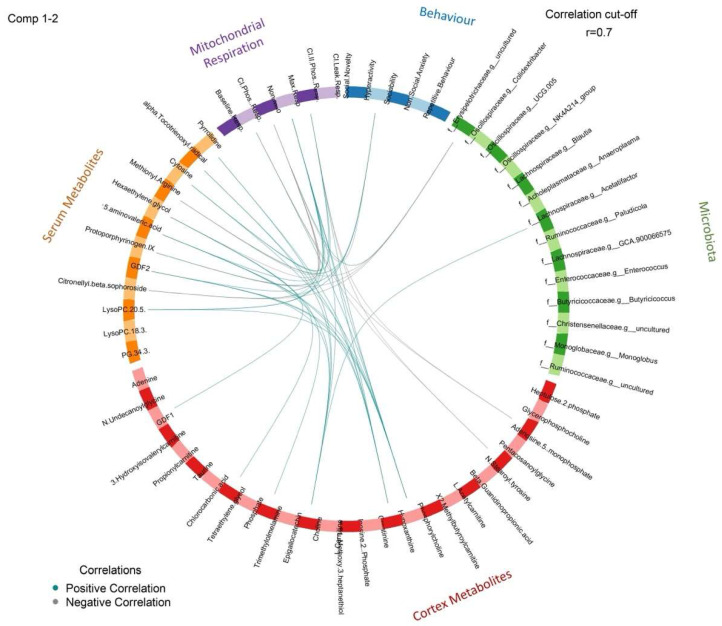
Circos plot showing significant correlations between gut microbial, metabolite, mitochondrial respiration, and behavioural data. A correlation cut-off of 0.7 was considered significant. Positive correlations are represented by blue lines and negative correlations are represented by gray lines.

To gain insight on relationships along the microbiota–gut–brain axis, we performed correlation analysis on significant gut microbial taxa, serum and cortex metabolites, mitochondrial respiration, and behavioural parameters. The circus plot in Figure 8 provides a summary of the correlations (|r| > 0.7) among the various key outcomes in this study. A heatmap summarizing these correlations can also be seen in Supplementary Figure S1.

## 4. Discussion

Interest in the use of *Lactobacillus* as an adjuvant treatment for neurodevelopmental disorders has increased significantly within the past decade [51]. In the current study, we evaluated the ability of two potential psychobiotic strains, *L. rhamnosus* HA-114 and *L. salivarius* HA-118, to mitigate ASD-related behaviours when administered in early life, a critical developmental window in which changes to the gut microbiota can have long-term health outcomes [52]. Specific effects of probiotic administration on the gut microbiota, systemic metabolic parameters, and mitochondrial metabolism were assessed to further understand changes along the microbiota–gut–brain axis.

Improvement in ASD-related behaviours is a key indicator of successful psychobiotic action. Changes in behaviour were recorded in the BTBR *T*^+^ *Itpr3^tf^*/J (BTBR) mouse strain, a validated preclinical rodent model of core ASD symptoms [53]. Our data illustrated that *L*. *rhamnosus* and *L. salivarius* supplementation reduced behavioural deficits associated with social novelty interaction in the BTBR mice, which can be related to the avoidance of stranger interactions observed in ASD children [54,55]. However, no changes were observed in hyperactivity, anxiety-like behaviour, repetitive/restricted behaviour, nor sociability following probiotic treatment. Despite the mounting evidence that *L*. *rhamnosus* supplementation improves a range of behavioural parameters [56,57,58], discrepancies in bacterial strains, length of treatment, host genetics, and age during treatment could be contributing to the modest changes in behaviour we observed in our study. Interestingly, little to no evidence exists in the literature supporting the beneficial effects of *L. salivarius* on behaviour, warranting further investigation into the potential role of this bacterial strain on behaviour. Additional research evaluating different methods of delivery, the use of single-strain versus combination probiotics, and optimal dosage of these specific probiotic strains could be used to enhance therapeutic efficacy. 

The use of probiotics to improve intestinal dysbiosis associated with ASD has gained significant traction in recent years. Proposed mechanisms of probiotics involve modulating the composition and function of gut microbial communities [59]. Our results show that *L*. *rhamnosus* alone increased microbial richness and phylogenetic diversity, as calculated by the Observed OTU and Faith’s Phylogenetic Diversity indices, respectively. Unweighted, but not weighted, Unifrac analysis revealed that *L*. *rhamnosus* treatment produced distinct microbial communities from the control and *L. salivarius* groups. As such, it is suggested that differences in microbial communities between our groups are mainly determined by bacteria of lower abundance in our samples. Given this, *L. rhamnosus* displays therapeutic potential in improving reduced microbial diversity, a phenotype that is frequently observed in human cohorts and animal models of ASD [60,61,62,63]. Furthermore, *L*. *rhamnosus* may improve overall performance along the microbiota–gut–brain axis, as a diverse gut has been associated with the regulation of GI barrier function and blood–brain-barrier permeability [64]. While *L. salivarius* did not alter microbial community structure or diversity, it could have had potential secondary effects on microbial cross-feeding, in which its metabolites facilitate interspecies interactions in the gut and may even have direct effects on the host [65]. These factors highlight the varying influences of *L. rhamnosus* and *L. salivarius* on the gut microbiota, which may underlie the amelioration of social novelty interaction behaviours observed following treatment. 

Given that *L*. *rhamnosus* supplementation led to discrete microbial clustering, specific taxa that contributed to this outcome were assessed. Following *L. rhamnosus* treatment, *Anaeroplasma*, *Acetatifactor, Lachnospiraceae*, *Christensenellaceae*, *Enterococcus,* and *Oscillospiraceae* were increased, while *Blautia* and *Butyricicoccus* were decreased, compared to controls. A species within *Anaeroplasma* has been implicated as a potential probiotic for chronic inflammation due to its ability to induce the anti-inflammatory cytokine transforming growth factor-β, ultimately enhancing the GI barrier by increasing mucosal IgA levels [66]. Similarly, *Christensenellaceae* also exhibits potential anti-inflammatory properties, as this taxon has been consistently depleted in conditions associated with inflammation (i.e., Crohn’s disease, ulcerative colitis) [67]. As a strong inflammatory state has been associated with ASD, increases in these anti-inflammatory taxa may aid in ameliorating ASD behavioural symptoms, as well as inflammation-induced GI comorbidities [68]. 

To determine the potential effects of probiotic administration on systemic inflammation, we assessed serum cytokine levels following treatment. *L. rhamnosus* significantly increased levels of IL-6, IL-10, IL-12p70, and IL4. Interestingly, studies assessing the cytokine composition in ASD patients identified elevated levels of IL-6 and IL-12p70 to be associated with pro-inflammatory action, potentially leading to neuroinflammation-related behavioural abnormalities [69,70]. However, levels of the anti-inflammatory cytokine IL-10 [71], shown to have important regulatory functions in neuroimmune responses, were also found to be decreased in autistic patients [72]. Thus, while we cannot confidently conclude whether *L. rhamnosus* promotes or inhibits an inflammatory profile, our results suggest that it may exhibit immunomodulatory effects through altering cytokine production. Further assessment of a complete cytokine screen regulated by L. *rhamnosus*, overlayed with evaluation of the interaction between the affected cytokines and target immune cells, is warranted to provide further insight into potential pro- or anti-inflammatory mechanisms of action. 

*Acetatifactor*, *Lachnospiraceae*, and *Butyricicoccus* are all butyrate-producing bacteria, indicating that levels of this short-chain fatty acid may be altered in response to *L. rhamnosus* treatment. In fact, this finding is supported by the significant increase in the acetyl-CoA pathway of butyrate production observed in the *L. rhamnosus* group. Unfortunately, short-chain fatty acids were not quantified in our metabolomics analyses. Butyrate activity has been shown to regulate inflammatory and immune processes, mainly through the suppression of nuclear factor kappa B and the activation of free fatty-acid receptors [73,74]. Furthermore, butyrate can support energy metabolism and mitochondrial function through the enhancement of oxidative phosphorylation and fatty-acid oxidation processes [75]. Interestingly, Rose and colleagues [76] suggested that butyrate over-production may be the driving force behind overactive mitochondria in ASD, a phenotype associated with increased vulnerability to oxidative insults. Consequently, further investigations into the effects of butyrate on energy metabolism and inflammation in BTBR mice would provide more clarity as to whether this short-chain fatty acid could be contributing to or mitigating ASD symptomology. 

A mechanism in which the microbiota is thought to exert its influence on neural function is through the production of bioactive metabolites, resulting in altered metabolic profiles. To determine potential mediators along the microbiota–gut–brain axis, we completed comprehensive metabolomics profiling on serum and prefrontal cortex tissue. This proposed mechanism was supported by the altered serum and brain metabolite compositions following *L. rhamnosus* administration, suggesting that this strain may influence metabolic profiles. Intriguingly, *L. rhamnosus* treatment resulted in an increase in multiple neuroactive compounds, including 5-aminovaleric acid (5-AV) and choline. To expand, 5-AV is a weak GABA receptor agonist that has been found to be significantly lower in individuals with ASD [77]. The administration of 5-AV to BTBR mice was sufficient to ameliorate the social deficits associated with autism, which was thought to work by potentially attenuating the excitatory/inhibitory imbalance that has been previously implicated in ASD pathophysiology [49]. In terms of choline metabolism, the social and behavioural abnormalities present in ASD symptomology have been associated with cholinergic pathways [78]. Perinatal supplementation of choline, as well as the inhibition of acetylcholine breakdown, a neurotransmitter in which choline is a precursor, reduced deficits in social and repetitive/restricted behaviours in BTBR mice [79,80]. Curiously, *L. rhamnosus* treatment also led to elevated levels of lysophosphatidylcholine (LysoPC) in serum, a key compound that supplies choline to the brain [81]. Furthermore, circulating LysoPC may have a protective role in neurodegenerative disorders, such as Alzheimer’s disease, as it is the preferred carrier of polyunsaturated fatty acids across the blood–brain barrier, which is critical for the maintenance of neuronal cell membranes [82]. Thus, our study demonstrated that influencing the production of neuroactive metabolites may be one mechanism to how *L. rhamnosus* HA-114 acts upon the microbiota–gut–brain axis. The potential relationships between metabolites, gut microbiota, and behaviour are also demonstrated in Figure 8; however, it is important to recognize that these associations are correlative and not causative. 

In addition to the regulation of neuroactive metabolites, changes in mitochondria-related metabolites were observed in the brain. *L. rhamnosus* treatment resulted in decreases of L-acetylcarnitine and adenosine 5-monphosphate, metabolites known to stimulate oxygen respiration in the mitochondria [83,84]. A substantial number of ASD cases exhibit mitochondrial dysfunction through the overactivity of the electron-transport chain, in which further stimulation of oxygen respiration would be unfavourable [22]. Although no significant differences in mitochondrial respiration rates were observed between our groups, a decrease in respiration-stimulating metabolites may work to balance energy metabolism in ASD individuals presenting with mitochondrial overactivity. Despite this, further investigations into mitochondrial respiration and electron-transport-chain activity are required to confirm these inferences.

Herein, we would like to acknowledge certain limitations of our study. While the treatment duration of 4 weeks for probiotic delivery surpassed previous reports in the BTBR mouse model [26], it may take longer to observe CNS-related effects, as the probiotic was dissolved in drinking water. As such, oral gavage may provide a more potent and accurate method for probiotic delivery in the future. Furthermore, while increases in serum levels of neuroactive metabolite 5-AV were observed following *L. rhamnosus* treatment, our study did not confirm its characterization in the brain. As we only assessed prefrontal cortex tissue, it would be beneficial to see if 5-AV was affected in other relevant brain areas; however, 5-AV cannot be directly related to the behavioural changes we observed in our data until this is demonstrated. Lastly, our assessment of circulating cytokines following *L. rhamnosus* treatment was not extensive, as only 10 cytokines were evaluated, leading to inconclusive results regarding the characterization of an inflammatory phenotype. A complete cytokine screen encompassing an extensive range of cytokines will shed more light on the potential contributions of *L. rhamnosus* on inflammation in ASD. Additionally, investigating alterations in immune signalling pathways may help to discern specific immunological responses affected by the identified cytokines.

## 5. Conclusions

The present study demonstrated the psychobiotic potential of *L. rhamnosus* HA-114 through the positive modulation of social interaction, gut microbial diversity, and neuroactive signalling molecules along the microbiota–gut–brain axis. Although *L. salivarius* HA 118 demonstrated positive effects on social behaviour, it had minimal impacts on the gut microbiota as well as neuroactive metabolites. Overall, *L. rhamnosus* HA-114 shows promise as a potential therapeutic treatment for ASD symptomology. We recommend further investigations into the downstream effects of *L. rhamnosus* administration on immune responses, neuronal activity, energy metabolism, and GI function to gain a better understanding of its mechanisms of probiotic action in ASD.

## Data Availability

The 16S rRNA gene-sequences data supporting the conclusions of this article are deposited at the National Center for Biotechnology Information (BioProject PRJNA 823373).

## Supplementary Materials

The following supporting information can be downloaded at: https://www.mdpi.com/article/10.3390/brainsci12060781/s1, Table S1: Substrates used to assess various mitochondrial respiratory states in the prefrontal cortex and hippocampus tissue of BTBR mice; Figure S1: Heatmap showing significant correlations between gut microbial, metabolite, mitochondrial respiration, and behavioural data.
