# Supplementary material for: Selective Probiotic Treatment Positively Modulates the Microbiota–Gut–Brain Axis in the BTBR Mouse Model of Autism
Source: Brain Sci. 2022 Jun 14;12(6):781. doi: 10.3390/brainsci12060781 (PMC9220969; doi:10.3390/brainsci12060781)
Supplement: Supplementary file 1 [file brainsci-12-00781-s001.zip › brainsci-1722503-supplementary.pdf]

Table S1. High-resolution respirometry was performed using an Oroboros Oxygraph-2K (Oroboros Instruments, Austria), which enables interrogation of mitochondrial function in intact tissue. Following dissection, 2 mg of prefrontal cortex and hippocampus was quickly weighed and transferred into calibrated Oxygraph-2K chambers containing MiR05 respiration medium. Following saponin permeabilization, the final substrate-inhibitor-uncoupled titrations were employed. Injections were performed in the same order as listed here. Oxygen concentration and oxygen flux per tissue mass (pmol $O2.s{-}1{\cdot}mg{-}1$) were recorded using DatLab software Oroboros Instruments, Austria).

**Substrates**	**Final Concentration**	**Function**
Saponin	20 mM	Plasma membrane permeabilization
Pyruvate	5 mM	NADH-generating substrate
Malate	2 mM	NADH-generating substrate
Glutamate	10 mM	NADH-generating substrate
Adenosine diphosphate (ADP)	2. 5 mM	Substrate of ADP/ATP translocase (ANT) and ATP synthase
Cytochrome C	10 µM	Test integrity of outer mitochondrial membrane
Succinate	10 mM	Substrate of Complex II
Carbonyl cyanide p-trifluoro-methoxyphenyl hydrazone (FCCP)	1 µM (0. 5µM steps)	Uncoupler and protonophore; induce maximum oxygen flux to determine ET capacity
Rotenone	0. 5 µM	Inhibitor of Complex I and NADH oxidation
Antimycin A (Ant)	2. 5 µM	Inhibitor of Complex III

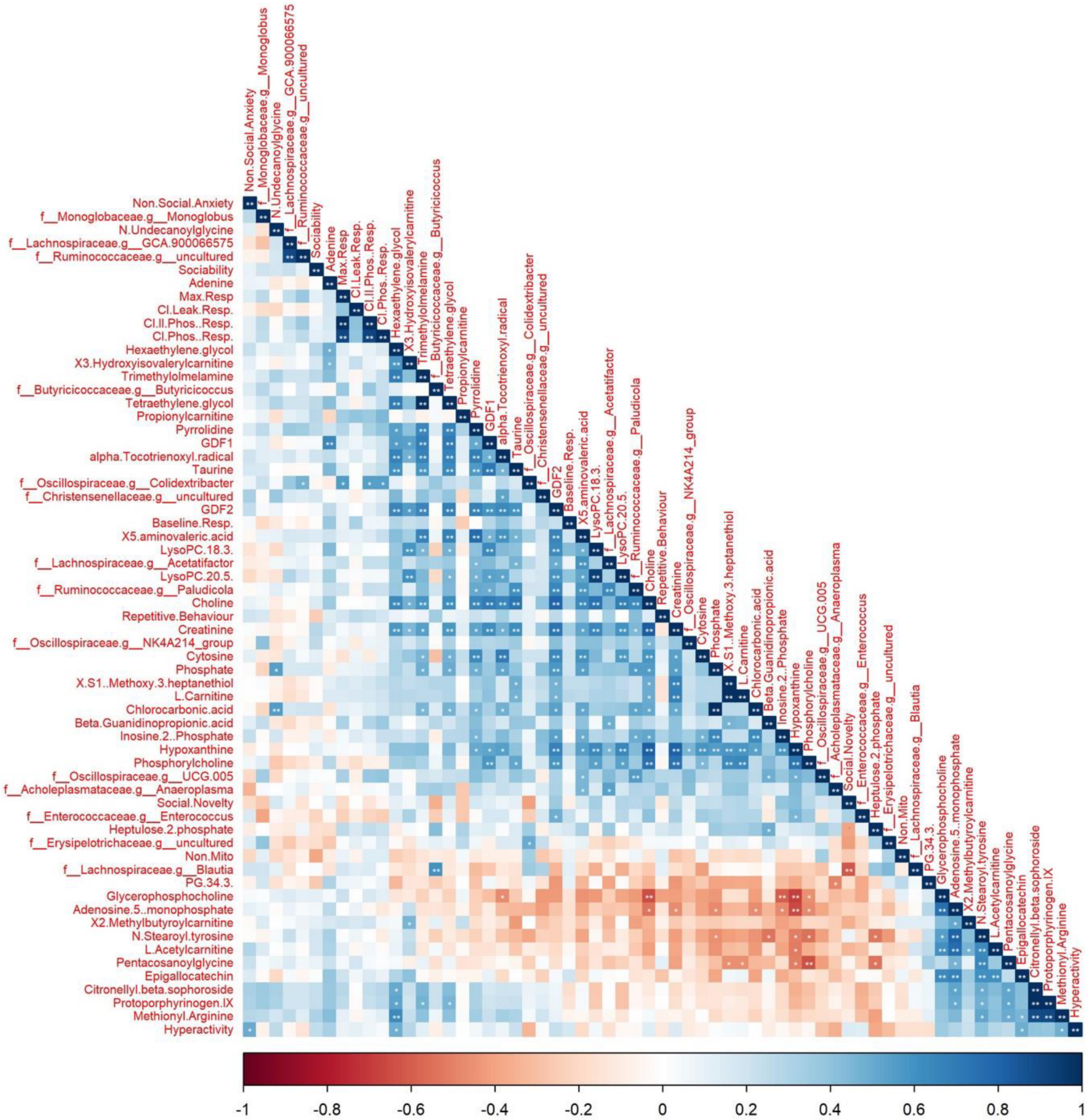

Figure S1. Heatmap showing significant correlations between gut microbial, metabolite, mitochondrial respiration, and behavioural data. Box colours represent Pearson's coefficient (r) value. *$p < 0.01$, **$p < 0.001$.
